# Paradoxical Progressive Multifocal Leukoencephalopathy With Immune Reconstitution Inflammatory Syndrome in a Patient With AIDS: A Case Report

**DOI:** 10.7759/cureus.92745

**Published:** 2025-09-19

**Authors:** Ibtisam A Ibrahim, Priscila Lopez, Vel Sivapalan

**Affiliations:** 1 Infectious Diseases, NYC Health + Hospitals/Harlem, New York, USA; 2 Infectious Diseases, Columbia University Irving Medical Center, Vagelos College of Physicians &amp; Surgeons, New York, USA

**Keywords:** demyelinating central nervous system (cns) disease, haart resumption, immune reconstitution inflammatory syndrome (iris), john cunningham virus (jcv), progressive multifocal leukoencephalopathy (pml)

## Abstract

Progressive multifocal leukoencephalopathy (PML) is a rare but often fatal demyelinating disease of the central nervous system, caused by the reactivation of the John Cunningham virus (JCV). It predominantly affects individuals with compromised immune systems, especially those living with AIDS. Immune Reconstitution Inflammatory Syndrome (IRIS) is a paradoxical event that can occur in patients receiving antiretroviral therapy (ART), where the immune system's recovery triggers a damaging inflammatory response. We present the case of a 39-year-old female patient with a history of AIDS and PML who was recently restarted on ART and presented with worsening left-sided weakness. She was managed for paradoxical PML and IRIS, and received steroids along with ART, with significant improvement.

## Introduction

Progressive multifocal leukoencephalopathy (PML) is an opportunistic, fatal viral disease in immunocompromised individuals that affects the central nervous system (CNS) [1-3], leading to a demyelinating disease of subcortical white matter due to the lysis of oligodendrocytes [1]. PML was first described by Astrom in 1958 and later linked to the John Cunningham virus (JCV) by Padgett in 1971 [2], now called human polyomavirus 2 (HPyV-2), a member of the Polyomaviridae family [1,2]. 

The incidence among the general population remains 0.22 per 100,000, despite the widespread use of antiretroviral therapy (ART) [4]. Although an HIV infection remains responsible for about 80% of new PML cases, the incidence associated with therapeutic monoclonal antibodies, such as natalizumab, efalizumab, and rituximab, is increasing [3]. 

PML may present with motor deficits (monoparesis or hemiparesis), cognitive impairment, limb or gait ataxia, visual disturbances (such as hemianopsia or diplopia), and speech difficulties. Less commonly, patients may experience headaches, seizures, or sensory loss [1]. Usually, the diagnosis is based on the clinical presentation, brain imaging findings (preferably magnetic resonance imaging or MRI), and the detection of the virus in the cerebrospinal fluid (CSF) by polymerase chain reaction (PCR) [1,2]. A definitive diagnosis of PML is made through brain biopsy, using either in situ hybridization to detect JCV DNA or immunohistochemical staining for JCV [1].

There is no antiviral drug available against JCV [5], and the sole therapeutic approach to PML involves initiating combined antiretroviral therapy (cART) in HIV-infected patients or discontinuing immunosuppressive agents in non-HIV-infected individuals. [3,4]. Mirtazapine, a serotonin receptor antagonist, has been reported to yield favorable outcomes in case studies in certain patients with PML; however, current evidence remains insufficient to support its efficacy [1]. Recent studies have shown that pembrolizumab, a humanized monoclonal antibody targeting the programmed cell death protein 1 (PD-1) receptor on lymphocytes, can reduce JCV viral load, enhance CD4+ and CD8+ T-cell responses against JCV, and lead to clinical stabilization or improvement in up to 62.5% of patients with PML [1].

It is important to remember that immune restoration is not invariably beneficial, as 16.7% of the HIV-infected patients with PML experience clinical deterioration following cART initiation, attributed either to severe neuroinflammation in the context of immune reconstitution inflammatory syndrome (IRIS) [1,3,6] or to an overwhelming inflammatory response against a pre-existing antigen or pathogen [6].

IRIS is characterized by immune reconstitution, reflected by rising CD4+ T-cell counts or declining plasma HIV RNA levels, accompanied by a marked worsening of neurological signs and symptoms [6]. 

The paradoxical form can cause confusion, making it unclear whether symptoms reflect PML progression, overlap with IRIS, or result from highly active ART's (HAART) adverse effects, toxicity, or failure. IRIS should be suspected when PML symptoms worsen or emerge four to eight weeks after starting HAART, especially if the CD4+ count is below 100 cells/mm^3^ before treatment, or if neurological symptoms develop or worsen during HAART [7]. 

PML-IRIS is classified as unmasking if the patient presents with new-onset neurological manifestations, or paradoxical if neurological manifestations are exacerbated after the initiation of ART [8]. 

For mild-to-moderate IRIS symptoms, symptomatic treatment such as analgesics, antipyretics, and non-steroidal anti-inflammatory drugs (NSAIDs) is usually effective. Systemic corticosteroids are the most commonly used and studied treatment for severe IRIS symptoms, despite their disadvantages [1]. 

## Case presentation

A 39-year-old female patient with a past medical history of anemia of chronic disease, HIV diagnosed 20 years ago, and non-adherent with antiretroviral medication, was restarted on ART two months ago when she sought medical care due to neurological symptoms. She was hospitalized for the symptoms and managed for PML. At that time, the patient presented with dysarthria and left-sided numbness and weakness, some memory lapses, and affective symptoms that were initially managed as adjustment syndrome. As a result, she was started on a selective serotonin reuptake inhibitor (SSRI). Laboratory testing was significant for JCV Ab serum positivity, JCV DNA by polymerase chain reaction (PCR) was 24,800 copies/ml in cerebrospinal fluid (CSF), HIV viral load was 23700, and CD4 count was 20 (Table 1).

**Table 1:** Laboratory studies CD4: Cluster of differentiation; WBC: White blood cell; BUN: Blood Urea Nitrogen; Ag: Antigen, IgM: Immunoglobulin M; JC: John Cunningham; MTB: Mycobacterium tuberculosis; PCR: Polymerase Chain Reaction; Ab: Antibody; CSF: Cerebrospinal fluid; EBV: Epstein-Barr Virus; VDRL: Venereal disease research laboratory.

Component	First admission	Current admission	Reference range and Units
Absolute CD4	20	not tested	489-1457 cells/uL
% CD4	2	not tested	30-62 %
HIV viral load	23,700	52	<=20 copies/mL
WBC	2.6	4.05	4.80-10.80 x10^3^/mcL
Hemoglobin	12.1	11.7	12.0-16.0 g/dL
Hematocrit	37.4	35.4	37.0-47.0%
Platelets	105	124	150-450 x10^3^/mcL
Creatinine	0.6	0.7	0.5-0.9 mg/dL
BUN	10	10	7-18 mg/dL
Cryptococcal Ag Serum	negative	negative	negative
Toxoplasma IgM Screen		<3.00	<=7.90 AU/mL
Acid-fast bacilli, sputum	No acid-fast bacilli isolated after 6 weeks.		No acid-fast bacilli
MTB PCR	not detected		Not detected
JC Virus PCR, Plasma	433		Not detected (copies/mL)
Treponema Pallidum Ab Screen I	negative		negative
Myelin Basic Protein	7.2		
Fungus CSF	No fungus isolated at 4 weeks		
Acid fast CSF	No acid-fast bacilli isolated after 6 weeks.		
Oligoclonal band, CSF	Present		
Fungitell B-D-Glucan, CSF	<60		
EBV PCR Result	not detected		
Cryptococcal Ag CSF	negative		
Protein, CSF	41		
Glucose CSF	54		
VDRL Spinal Fluid	Non reactive		
JC Virus DNA by PCR, CSF	24,800		
Quantiferon Plus Tb	Negative		
Cytomegalovirus PCR	Not detected		

**Figure 1:**
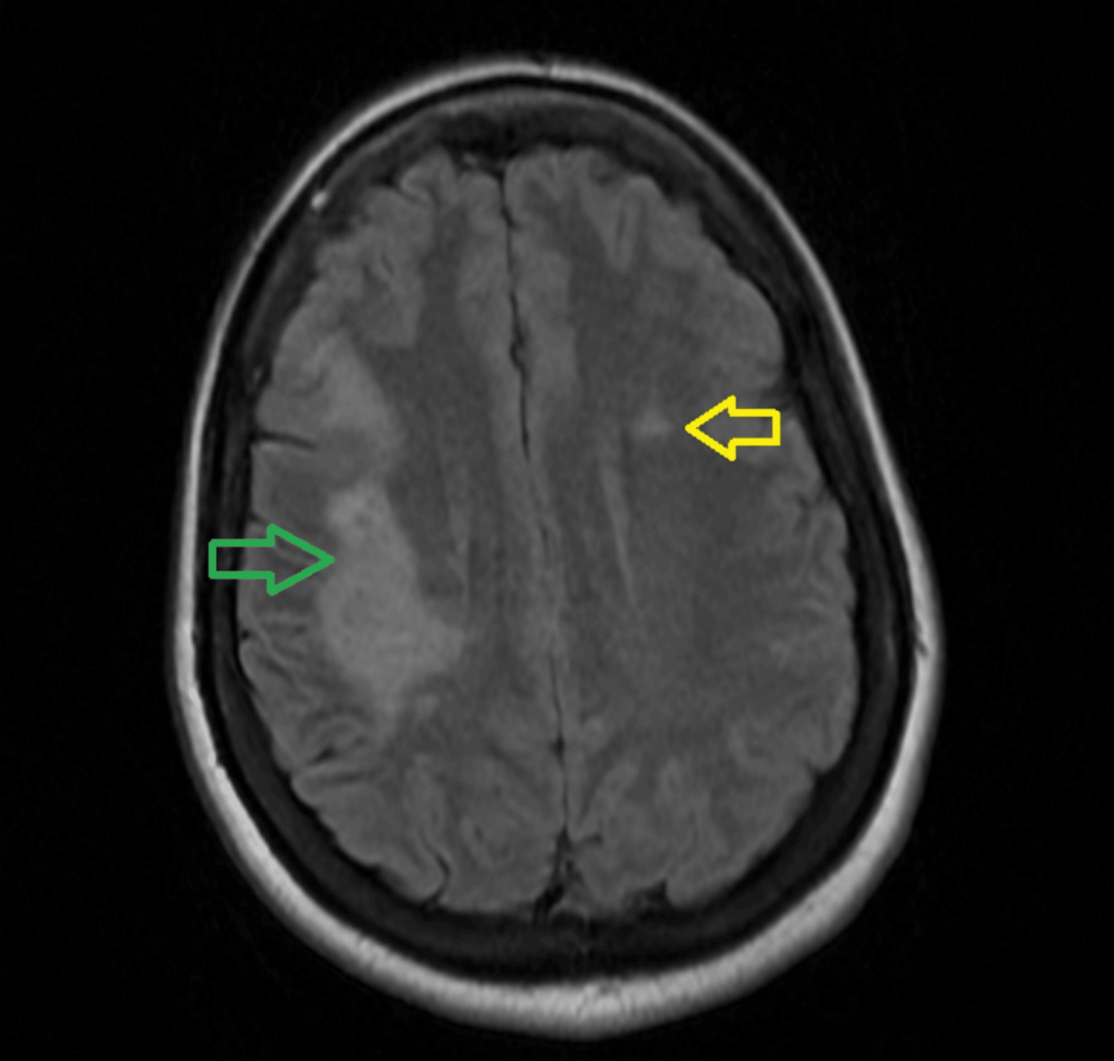
: Initial MRI brain with and without contrast - Axial T2 FLAIR FLAIR:  Fluid attenuated inversion recovery. The green arrow indicates a moderate area of hyperintensity within the subcortical, periventricular, and deep white matter of the right frontal and parietal lobes. The yellow arrow indicates a  small, nonspecific foci of increased T2/FLAIR signal in the left frontal and periventricular white matter

MRI brain with and without contrast revealed hyperintensities in the frontal and parietal white matter (Figure 1).

The patient was readmitted with worsening of the left side numbness, weakness, and dysphagia without odynophagia. On physical examination, she was hypertensive (138/105 mmHg), with the rest of the vital signs stable, temperature 98.1° F, pulse 95 bpm, respiratory rate 18 bpm, saturation 100% on room air. Significant neurological findings positive for left facial droop, left upper extremity motor 0/5, left lower extremity 3/5, right upper and lower extremity motor 5/5, sensation intact.

Laboratory testing was significant for HIV viral load (52), whereas cell blood count and chemistry metabolic panel were within normal limits (Table 1). Initial imaging of CT head revealed a large hypodense area within the right frontal and right parietal lobe subcortical white matter involving the right frontal lobe centrum semiovale and corona radiata, which had increased in size compared to the prior study (Table 2).

**Table 2:** Imaging MRI: Magnetic Resonance Imaging; T1/T2: Image sequences; FLAIR: Fluid-Attenuated Inversion Recovery

Timing	Imaging	Report
Previous admission	MRI brain with and without contrast	Area of moderate area of abnormal T2/FLAIR signal in the right frontal and parietal white matter with possible faint peripheral enhancement (Figure 1)
This admission	MRI brain without contrast	Marked increase in size and extent of the previously noted T2/FLAIR hyperintense lesion with T1 hypointensity within the periventricular, deep and subcortical white matter of the right frontal and parietal lobes, with interval development of a similar, moderate-sized T2/FLAIR hyperintense lesion with tiny internal T1 hypointense focus, within the corresponding white matter of the left frontal lobe. There was an associated sulcal effacement in both cerebral hemispheres as described, likely related to moderately prominent local mass effect bilaterally (Figure 2)

**Figure 2:**
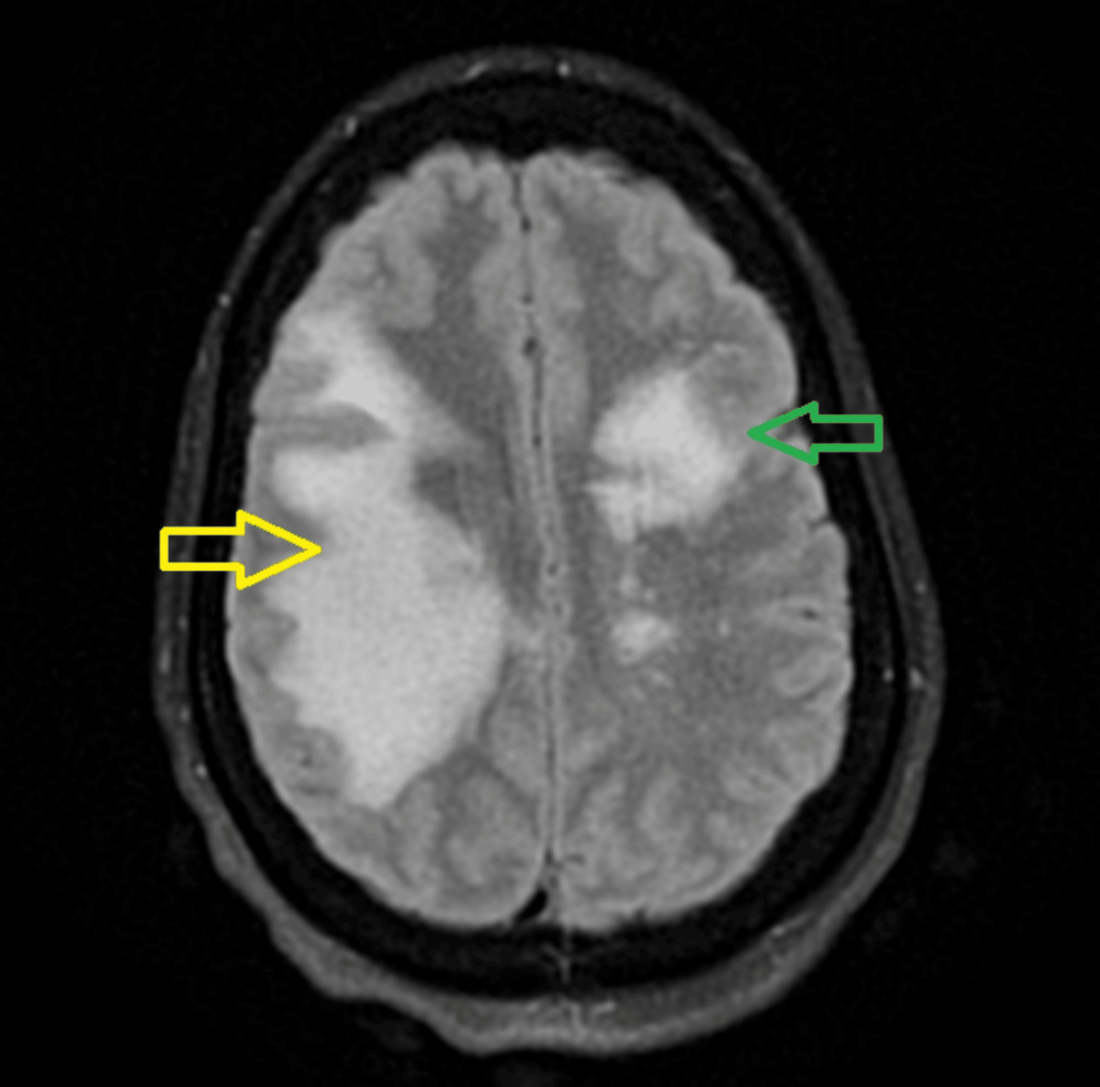
New MRI brain with and without contrast – Axial T2 FLAIR FLAIR: Fluid-Attenuated Inversion Recovery. The yellow arrow indicates a marked increase in the size and extent of the hyperintense lesion of the right frontal and parietal lobes. The green arrow indicates an interval development of a similar, moderate-sized hyperintense lesion within the corresponding white matter of the left frontal lobe.

MRI of the brain without contrast revealed a marked increase in the size and extent of the previously noted T2/Fluid-Attenuated Inversion Recovery (FLAIR) hyperintense lesion with T1 hypointensity within the periventricular, deep and subcortical white matter of the right frontal and parietal lobes. There was also an interval development of a similar, moderate-sized T2/FLAIR hyperintense lesion with tiny internal T1 hypointense focus, within the corresponding white matter of the left frontal lobe. There was an associated sulcal effacement in both cerebral hemispheres as described, likely related to moderately prominent local mass effect bilaterally (Figure 2).

The patient was continued on HAART along with trimethoprim-sulfamethoxazole and managed with interdisciplinary teams, including neurology, who recommended methylprednisolone 1 g daily for three days, and to be continued for the next two days (total five days) following by a tapering dose of 1 mg/kg/day for two weeks.

The patient demonstrated improvement and was discharged home in a stable condition. She was encouraged to follow up with the Virology clinic as an outpatient.

## Discussion

This case reflects the complexity of managing PML and IRIS, highlighting the importance of timely recognition and treatment of both neurological and psychiatric symptoms.

The case exhibited clinical and radiological evidence of PML, complicated by IRIS. The inflammatory response is driven by the rapid restoration of immune function, resulting in an exaggerated immune activation against JCV-infected cells approximately seven weeks after resumption of HAART. The development of IRIS approximately seven weeks after HAART resumption is consistent with the reported timeline in the literature. According to Tan et al. [9], PML-IRIS develops between one week and 26 months after initiation of ART. IRIS typically manifests within four to eight weeks of initiating HAART, with a median onset of six weeks [10]. 

PML is a demyelinating disease of the central nervous system (CNS) resulting from reactivation of JCV, which produces a lytic infection of oligodendrocytes. Primary JCV infection typically occurs during childhood and is asymptomatic, after which the virus persists in a latent state, possibly within lymphoid organs, neuronal tissue, or the kidneys [11]. IRIS is defined as paradoxical worsening of a patient’s clinical condition that is attributed to the recovery of the immune system after initiation of combined ART [12]. 

There is currently no consensus on a specific treatment strategy for PML-IRIS in patients with AIDS beyond HAART. Although HAART itself can precipitate IRIS by restoring immune function, it remains the cornerstone of treatment, as immune reconstitution is essential for controlling JCV replication [8]. 

In patients of PML-IRIS previously treated with intravenous dexamethasone (32 mg daily in four divided doses) or intravenous methylprednisolone (1 g daily for five days) followed by tapering doses without interrupting ART, clinical improvements were observed, yet no difference in overall survival was noted [13]. 

The initial presentation in our case included left-sided weakness, numbness, and dysarthria. During IRIS, the motor and sensory symptoms observed in this patient, including left-sided weakness and numbness, were consistent with the neurological manifestations frequently reported in PML. According to McArthur et al. [14], motor deficits are among the most common presenting symptoms, occurring in approximately 50-75% of PML cases. Sensory symptoms, though less common, are also documented in 10-20% of cases. Dysarthria, as observed in this patient, reflects involvement of subcortical structures and is reported in 20-30% of cases [15]. Our patient developed additional cognitive symptoms, memory lapses, and worsening depressive symptoms. Notably, these depressive symptoms were initially misattributed to adjustment disorder and reactive emotional responses but later recognized as primary affective symptoms, prompting the initiation of mirtazapine. Mirtazapine’s potential benefits in PML, beyond its antidepressant effect, stem from in vitro evidence suggesting inhibition of 5-hydroxytryptamine (serotonin) receptor 2A (5-HT2A) receptors involved in the JCV infection of oligodendrocytes [15] and its suggested beneficial effects in PML progression from limited clinical data. However, this requires further validation through clinical trials.

Radiologically, the lesions predominantly involved the subcortical frontal and periventricular parietal regions, with imaging demonstrating lesion enlargement and extension suggestive of IRIS despite the absence of contrast. The radiological findings in this case, frontal and parietal involvement, align with Zięba et al. [16], who reported similar patterns in 60-70% of cases. However, other brain regions such as the occipital lobe and cerebellum, which are affected in 10-20% of cases, were not involved in this case. 

Tapering corticosteroid therapy resulted in clinical improvement in dysarthria, numbness, and cognitive symptoms, and stabilization of motor weakness. Steroid therapy effectively mitigated IRIS-related inflammation in this case, as evidenced by stabilization or improvement of neurological symptoms. The utility of corticosteroids in managing PML-IRIS has been reported in several studies [5,12]. McArthur et al. [14] observed clinical improvement in 65-75% of PML-IRIS cases treated with steroids, particularly in reducing inflammation-mediated symptoms such as cognitive deficits and motor impairments. Management strategies for patients with PML-IRIS involve the use of corticosteroids and maraviroc; however, the supporting evidence remains largely anecdotal due to the lack of clinical trials [1,3]. While corticosteroids help reduce inflammation, they may also expand the pool of HIV-infected cells, thereby hindering JCV clearance [17]. The management of PML-IRIS in patients with AIDS primarily involves HAART, though its effectiveness in achieving full remission remains uncertain [18]. Additional treatment approaches, such as corticosteroids and maraviroc, have been explored, but their efficacy is largely based on anecdotal evidence rather than robust clinical trials. This patient’s favorable response to corticosteroids reinforces their role in mitigating IRIS-related inflammation. 

## Conclusions

In conclusion, PML-IRIS is a complex and challenging condition that arises in HIV-infected patients undergoing ART, particularly after immune reconstitution. The management of PML-IRIS involves a combination of HAART, corticosteroids, and, in some cases, maraviroc, although clinical evidence for these treatments remains limited. Corticosteroids can alleviate inflammation and improve neurological symptoms, but their use may hinder JCV clearance and expand the HIV-infected cell reservoirs. While HAART remains the primary treatment, the lack of consensus on a specific therapeutic approach highlights the need for further clinical research. This case underscores the importance of early recognition and tailored treatment strategies for both the neurological and psychiatric symptoms associated with PML-IRIS.
